# Diagnostic value of SOX-10 immunohistochemical staining for the detection of uveal melanoma

**DOI:** 10.3332/ecancer.2015.566

**Published:** 2015-08-20

**Authors:** Sarah A Alghamdi, Pablo Zoroquiain, Ana Beatriz T Dias, Sulaiman R Alhumaid, Sultan Aldrees, Miguel N Burnier

**Affiliations:** Henry C Witelson Ocular Pathology Laboratory, McGill University, H4A 3J1, Canada

**Keywords:** uveal melanoma, SOX-10, immunohistochemistry

## Abstract

**Objectives:**

SOX-10 has been shown to be a sensitive marker of cutaneous melanoma. This study aimed to evaluate Sox-10 expression in uveal melanoma.

**Methods:**

A total of 40 tissue blocks of enucleated eyes with uveal melanoma were cut and stained using an anti-SOX-10 mouse monoclonal antibody and HMB-45 antibody.

**Results:**

SOX-10 showed exclusive nuclear positivity in 100% of the uveal melanoma cases (38/38). HMB-45 showed cytoplasmic positivity in 97.3 (37/38). Positivity for SOX-10 was also noted in the inner and outer nuclear layers of the retina in 78% of the enucleated eyes.

**Conclusions:**

SOX-10 expression proved to be the most sensitive marker for uveal melanoma, and therefore, we propose a modified panel for the diagnosis of uveal melanoma that includes both SOX-10 and HMB-45. The observation of distinct, diffuse nuclear SOX-10 expression in retinal inner and outer nuclear layers is a finding that warrants further investigation as a marker for retinoblastoma.

## Introduction

An immunohistochemical panel of S100 protein, Melan A and HMB-45 is commonly used to confirm the diagnosis of malignant melanoma due to the lack of adequate specificity and sensitivity of a single marker [1]. SOX-10 is a neural crest transcription factor that plays an important role in the specification of Schwann cells and melanocytes [2, 3]. SOX-10 has been shown to be a sensitive marker of cutaneous melanoma, including desmoplastic subtype, which is known to be negative for melanocytic markers such as Melan A and HMB-45 [4]. Compared to cutaneous melanoma, uveal melanoma differs in its biologic behaviour, metastatic potential, and therapeutic response [5]. To date, we are not aware of any study comparing the staining pattern of SOX-10 in uveal melanoma to the staining pattern of HMB45, which is considered to be the most sensitive marker for uveal melanoma in clinical practice [6]. This study aimed to evaluate SOX-10 expression in uveal melanoma.

## Methods

All data accumulation was obtained in accordance with Canadian and Quebec provincial legislation and the tenets of the Declaration of Helsinki. About 40 uveal melanoma cases over a period of 35 years (1980–2014) were retrieved from the Henry C. Witelson Ocular Pathology Laboratory; McGill University (Montreal, Quebec, Canada) on the basis of the availability of representative tissue. Cell type was classified according to the modified Callendar’s classification [7]. The cases were reviewed by two ocular pathologists to confirm the diagnosis.

Formalin-fixed, paraffin-embedded blocks of enucleated eyes with uveal melanoma were cut and stained using automated immunohistochemistry, performed using the Ventana benchmark machine according to the manufacturer’s recommended protocol (Ventana Medical Systems, Inc., Tucson, AZ, USA). This fully automated process involved barcode-labelling the slides, baking the slides, solvent-free deparaffinisation, and CC1 (Tris-EDTA buffer [pH 8.0])-based antigen retrieval. Slides were incubated with an anti-SOX-10 mouse monoclonal antibody (Sox-10 (A-2): sc-365692) at a dilution of 1:600 for 30 minutes at 37°C followed by the application of a biotinylated secondary antibody for 8 minutes at 37°C, and then, an avidin/streptavidin enzyme conjugate complex was added. Finally, Fast Red was used as the chromogenic substrate and slides were counterstained with haematoxylin. Negative controls were performed omitting the primary antibody. Cutaneous melanoma was used as a positive control. For HMB-45, a mouse monoclonal HMB-45 antibody was used (HMB-45: 790-4366, Ventana validated protocol). The staining of both, SOX-10 and HMB-45, was scored based on the extent of the nuclear and cytoplasmic expression, respectively: diffuse when staining was positive in more than 50% of cells, and focal when it was seen in less than 50% of the cells.

## Results

**Figure 1.**
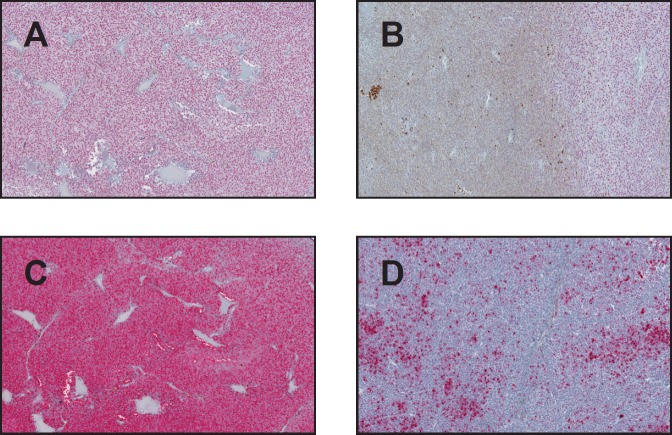
SOX-10 and HMB-45 expression in uveal melanoma (original magnification X400 [A through D]) (A) Diffuse nuclear SOX-10 positivity (B) Focal SOX-10 staining (C) Diffuse HMB-45 cytoplasmic staining pattern (D) Focal HMB-45 positivity.

**Figure 2.**
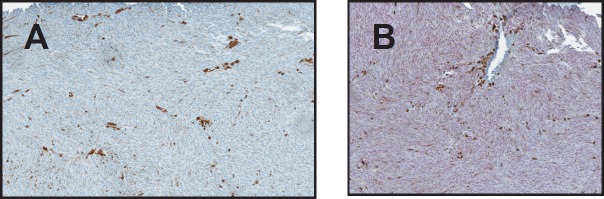
(A) Negative HMB-45 in a case of uveal melanoma (B) SOX-10 showing diffuse strong positivity of the same case. (original magnification X400 A & B).

**Figure 3.**
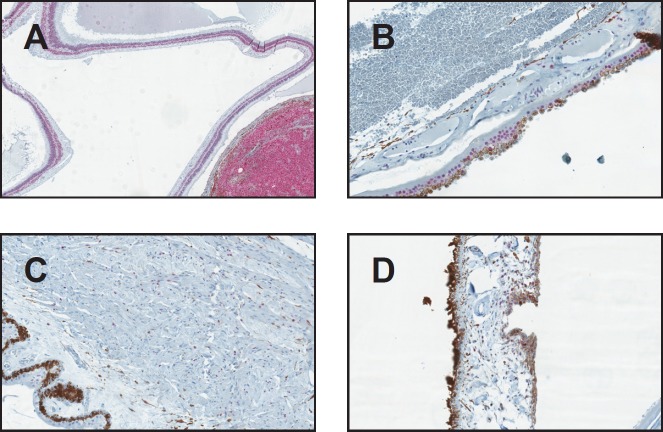
SOX-10 positivity in the uninvolved ocular structures (original magnification X400 [A through D]) (A) SOX-10 positivity in the nuclear layers of the retina (B) RPE and choroid showing focal SOX-10 positivity (C) Ciliary body showing scattered SOX-10-positive stromal melanocytes (D) Iris with scattered SOX-10 positive stromal melanocytes.

**Figure 4.**
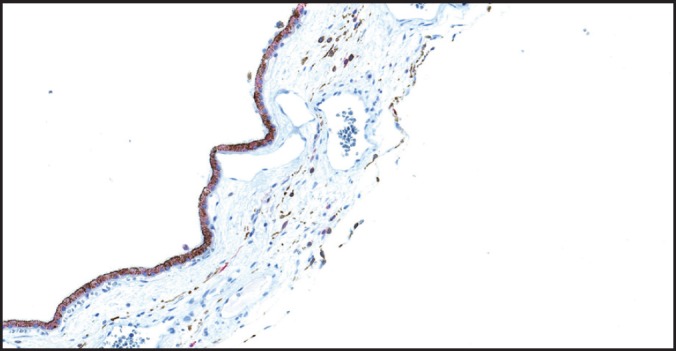
HMB-45 staining RPE cells and scattered melanocytes in the uninvolved choroid (original magnification X400).

From the 40 uveal melanoma cases reviewed, two cases were excluded because no viable cells were detected due to extensive necrosis. The remaining 38 uveal melanomas were categorised as follows: epithelioid (25%, *n* = 10), spindle (37.5%, *n* = 15), and mixed (32.5%, *n* = 13). The mean age of diagnosis was 69 with no gender predilection. SOX-10 showed exclusive nuclear positivity, as previously reported, in 100% of the uveal melanoma cases (38/38). Thirty-six showed diffuse nuclear positivity for SOX-10 (94.7%) (Figure 1A), while two had focal nuclear staining (5.3%) (Figure 1B). HMB-45 showed diffuse cytoplasmic positivity in 36 cases (94.7%) (Figure 1C), focal cytoplasmic staining in one (2.6%) (Figure 1D) and was negative in one case (2.6%), in which Sox-10 showed diffuse strong nuclear staining (Figure 2A, B). Positivity for SOX-10 was also noted in the inner and outer nuclear layers of the retina in 78% of the enucleated eyes (Figure 3A). Overall, 17% of the cases showed SOX-10 nuclear staining in the retinal pigmented epithelium (RPE) (Figure 3B). The stromal melanocytes were positive for SOX-10 in the uninvolved choroid, ciliary body, and iris in 44%, 48%, and 11%, respectively, (Figures 3B, C, and D). Five cases expressed SOX-10 in Schwann cells of the trans-scleral nerves (13%). HMB-45 showed focal positivity in the RPE cells in 39.5% of the cases and was positive in scattered stromal melanocytes in the uninvolved choroid, iris, and ciliary body in 53%, 8%, and 3% of the cases (Figure 4).

## Discussion

The most frequently used melanocytic markers in clinical practice are S-100 protein, HMB-45, and Melan-A [1]. While S-100 protein is highly sensitive for cutaneous melanomas, it shows less sensitivity with choroidal melanoma, staining about 50% of the cases [6, 8]. HMB-45 has a better sensitivity with choroidal melanoma, depending on the histologic subtype, ranging between 69–79% in spindle/mixed subtypes and 100% in epithelioid uveal melanoma [9]. Melan-A has a sensitivity similar to HMB-45 in choroidal melanomas [9]. SOX-10 has been shown to be a sensitive marker for cutaneous melanoma and is highly specific to distinguish melanoma from its non-melanocytic mimickers, such as excision scar, atypical fibroxanthoma and dermatofibrosarcoma protuberans [4, 10].

In this study, we sought to compare the utility of the most commonly used uveal melanoma marker HMB-45 to the recently described SOX-10. SOX-10 is a neural crest transcription factor important for the differentiation and commitment of Schwann cells and melanocytes [11]. To date, no published study has compared the SOX-10 staining pattern to the current, most reliable marker for uveal melanoma, HMB-45 in human enucleated eye samples [6]. Our results from 38 tumours indicate that SOX-10 provides a highly sensitive marker for uveal melanoma (38/38). Therefore, we suggest using a panel of SOX-10 and HMB-45 for diagnosing primary uveal melanoma. This concise panel replaces less sensitive markers, such as S-100 with SOX-10, and eliminates the redundant use of Melan-A, which has the same sensitivity as HMB-45 for uveal melanoma. Pathologists are infrequently exposed to specimens from the uveal tract, and uveal melanoma is a rare tumour that can be variably melanotic or even amelanotic, therefore, having a sensitive and specific panel can be of a great practical diagnostic utility to aid in reaching the right diagnosis [12, 13]. To the best of our knowledge, our study is the first to compare the expression of SOX-10 to HMB-45 in human bioptic uveal melanoma samples. However, in 2011, Kalirai *et al* were able to demonstrate the expression of SOX-10, as a primitive migratory neural crest marker, along with HMB-45 in human uveal melanoma cell lines from both the primary tumour (Mel270) and a cell line derived from a liver metastasis of the same patient (Omm2.5). Their results serve as an independent validation for the expression of SOX-10, along with HMB-45 in uveal melanoma [14].

**Figure 5.**
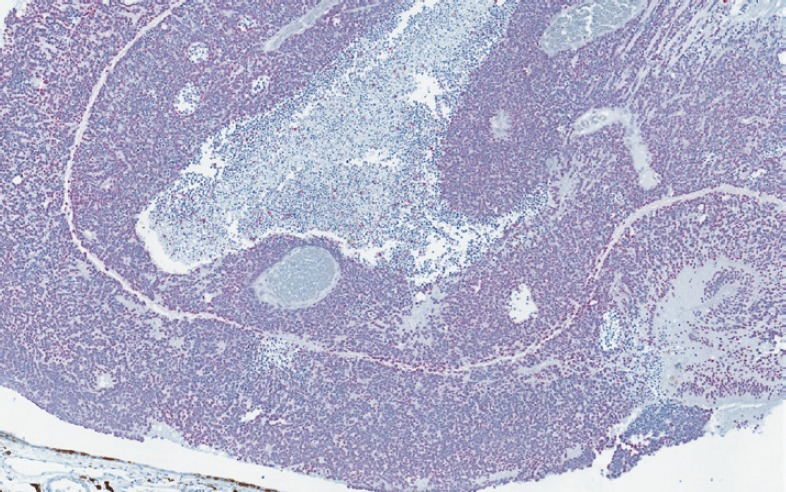
Retinoblastoma showing diffuse nuclear positivity for SOX-10 (original magnification X400).

This study documents, for the first time, the diffuse nuclear expression of SOX-10 in retinal inner and outer nuclear layers, a finding that warrants further exploration. We have also been able to demonstrate diffuse SOX-10 nuclear positivity in a case of retinoblastoma (Figure 5). Previous studies have demonstrated SOX-10 protein expression in human benign tissues including melanocytes, cranial ganglia, dorsal root ganglia, the otic vesicle, and myoepithelial cells of salivary, bronchial, and mammary glands [2, 15–17].

Although SOX-10 has been proven to be a superior marker for melanoma in skin, benign melanocytic lesions also express SOX-10; therefore, SOX-10 cannot be used to differentiate benign from malignant melanocytic skin lesions [16]. In ocular tissue, Sox-10 was positive in benign melanocytic component of the ciliary body, choroid, and iris. Therefore, SOX-10 cannot be used to differentiate melanoma from benign melanocytic tissue in ocular specimens. SOX-10 can be useful when analysing heavily pigmented uveal melanoma. In such cases, the clear nuclear signal of SOX-10 is easier to interpret when compared to the cytoplasmic staining of HMB-45 because of the extensive background pigmentation if bleaching method was not applied.

From a clinical point of view, the differential diagnosis for chroridal melanoma is metastatic carcinoma, particularly if the patient has a known primary. SOX-10 can potentially differentiate choroidal melanoma from metastatic carcinomas, since it has been proven to be negative in epithelial carcinomas [2]. Studying eyes from the autopsies of patients with uveal melanoma, along with other patients known to have carcinomas such as breast, colon, and lung, can provide a great objective method to demonstrate the utility of SOX-10 in these clinical scenarios.

Since alternative treatment modalities, such as brachytherapy (or external beam radiation), have recently increased aiming to preserve the eyes with uveal melanoma, enucleation rates have decreased [18]. With the recent advances in the molecular genetics of uveal melanoma, patients at high risk of metastasis can be identified early so that individualised management can be offered [19]. Taken together, the role of fine-needle aspiration (FNA) cytology specimens for diagnosing and classifying uveal melanoma is expanding. In such cases, using SOX-10 with its crisp nuclear profile offers a distinct advantage over the cytoplasmic staining of HMB-45. These FNA specimens may have retinal pigmented epithelial cells, which can be positive for SOX-10, mimicking uveal melanoma cells. In such scenario, the right diagnosis can be easily reached by adding a pan-cytokeratin immunohistochemical stain to the panel, which would be positive in the RPE cells and negative in uveal melanoma. In addition, the presence of dysplastic changes in uveal melanoma cells helps differentiate them from the RPEs cells.

## Conclusions

To the best of our knowledge, this is the first study investigating SOX-10 expression in uveal melanoma. SOX-10 expression proves to be the most sensitive marker for uveal melanoma; however, its specificity is less than optimal due to positivity in normal ocular structures. While SOX-10 has recently been described as a pan-schwannian and melanocytic marker with an excellent profile of sensitivity and specificity towards melanocytic lesions in the skin, its lack of specificity to melanocytes in ocular specimens leaves HMB-45 as the most specific marker for uveal melanoma. Therefore, we propose a modified panel for the diagnosis of uveal melanoma that includes both SOX-10 and HMB-45. Furthermore, the observation of distinct, diffuse nuclear SOX-10 expression in retinal inner and outer nuclear layers is a finding that warrants further investigation as a marker in paediatric tumours.
